# Face Reading

**Published:** 1896-04

**Authors:** 


### Face-Reading.

"In the acquisition of the art of speech-reading by sight," says *The Popular Science Monthly*, "the eye of the deaf pupil becomes accustomed to certain positions of the organs of articulation, and thus learns to understand the spoken words of others, although he does not hear them. In teaching this art, Lillie Eginton Warren has found that the forty odd sounds of the English language are revealed in sixteen outward manifestations or pictures, and practice in following them as they rapidly appear in a face enables us to understand what is said. Some faces differ from others in strength of expression, and thus many show less action in the lower part. Nevertheless, there is in all per sons a general approach to a certain definite movement of muscles, particularly when in animated conversation, and the trained eye notices what the inexperienced one fails to discover. After attaining a degree of proficiency in this art of expression-reading, persons seem to feel that they hear instead of see the words spoken. Reading our language in this way may be said to be mastery of a new alphabet, the rapidly moving letters or characters of which are to be found upon the page of the human countenance."—*Literary Digest.*

